# Open Partial Nephrectomy with Zero Ischaemia Using a Supra 12th Rib Miniflank Incision: A Minimally Invasive Open Approach for Small Renal Masses

**DOI:** 10.1155/2021/5569254

**Published:** 2021-12-31

**Authors:** Syed Ali Ehsanullah, Abida Sultana, Brian Kelly, Charlotte Dunford, Zaheer Shah

**Affiliations:** ^1^Worcestershire Acute Hospitals NHS Trust, Redditch, UK; ^2^University Hospitals Birmingham NHS Foundation Trust, Birmingham, UK

## Abstract

**Introduction:**

To assess a minimally invasive open technique for partial nephrectomy with zero ischaemia time.

**Methods:**

A review was performed in a prospectively maintained database of a single surgeon series of all patients undergoing partial nephrectomy using a supra 12^th^ rib miniflank incision with zero ischaemia. Data of seventy one patients who underwent a partial nephrectomy over an 82-month period were analyzed. Data analyzed included operative time, estimated blood loss, pre and postoperative renal function, complications, final pathological characteristics, and tumour size.

**Results:**

Seventy one partial nephrectomies were performed from February 2009 to October 2015. None were converted to radical nephrectomy. Mean operative time was 72 minutes (range 30–250), and mean estimated blood loss was 608 mls (range 100–2500) with one patient receiving blood transfusion. The mean pre and postoperative haemoglobin levels were 144 and 112 g/l. The mean pre and postoperative creatinine levels were 82 and 103 Umol/L. There were 8 Clavian–Dindo Grade 2 complications and 1 major complication (Clavian IIIa). Histology confirmed 24 benign lesions and 47 malignant lesions, 46 cT1a lesions, 24 cT1b lesions, and 1 cT2 lesion. Median follow-up was 38 months with no local recurrence or progression of disease with 5 patients having a positive margin (7%).

**Conclusion:**

Our results demonstrate that a supra 12th miniflank incision open partial nephrectomy with zero ischaemic time for SRMs has satisfactory outcomes with preservation of renal function. A minimally invasive open partial nephrectomy remains an important option for units that cannot offer patients a laparoscopic or a robotic procedure.

## 1. Introduction

There has been an increase over the past few decades of the incidental diagnosis of small renal masses (SRM) coinciding with the increasing use of computed tomography (CT) and magnetic resonance imaging (MRI). The diagnosis and treatment of small renal masses continues to evolve with advancements in imaging techniques and surgical approaches [1]. Nephron sparing surgery (NSS) or partial nephrectomy (PN) is now recommended by many international guidelines when patients elect to undergo surgical management of a small renal mass (SRM) [2, 3]. The management of SRMs has evolved over time from an open technique to minimal access approaches including laparoscopic and robotic-assisted techniques. Open nephrectomy (ON) was initially considered the “gold standard,” but laparoscopic and robotic approaches now have comparable oncological outcomes [2]. However, open PN still remains an important surgical option for high risk tumours.

The TRIFECTA criteria states that regardless of the technique employed, there should be a safe excision of the lesion, with minimal warm ischaemia time (<25 minutes) and no perioperative complications [4–6]. As such, clinical T1a lesions are best managed by PN, yet debate remains how best to manage larger renal masses. The European Association of Urology recommends PN for T1b lesions, while the American Association of Urology recommends a radical nephrectomy [2, 3]. The TRIFECTA criteria advocated a warm ischaemia time of less than 25 minutes. However, many would argue that “time is tissue” and that “every minute counts” to reduce the risk of long-term renal impairment [7, 8].

Our minimal access open technique includes insertion of a ureteric stent in all patients and a supra 12th rib flank incision of 6–8 cm without renal artery ischaemia for high-risk renal masses (e.g., with a high RENAL nephrometery scores). We present a series of 71 open partial nephrectomies.

## 2. Methods

Data were prospectively collected and maintained in our departmental database for a single surgeon series of partial nephrectomies. Data of 71 patients who underwent a partial nephrectomy over an 81/82 month period were analyzed (Feb 2009–Nov 2015). Data analyzed included operative time, estimated blood loss, pre- and postoperative renal function, complications as per Clavien–Dindo classification, final histopathological characteristics, and tumour size. Inclusion criteria were all cT1 and cT2 lesions which were amenable to PN. TNM was assigned according to the 2009 UICC Classification of Malignant Tumours 7th Edition version. Three histological subtypes were classed according to the World Health Organization (WHO) classification [9]. The nuclear grade was classified according to the Fuhrman grading system [10]. A positive surgical margin was defined as the presence of cancer cells at the level of the inked parenchymal excision surface.

All patients underwent a triphasic CT to assess both the renal and tumour anatomy. All patients were subjected to a preoperative assessment by an anaesthetist to assess suitability for surgery. Medical charts were reviewed for patient demographics, tumour characteristics, and intraoperative data. Patient demographic data included age, sex, BMI, ASA status, and comorbidities. Tumour characteristics included tumour size, tumour site, number of tumours, solitary kidney, and histological subtype. Intraoperative data included operative time, estimated blood loss, pelvicalyceal system repair, pre- and postoperative haemoglobin, and renal function. Postoperative complications were assessed and classified according to Clavien–Dindo's classification [11]. Statistical analysis was performed using SPSS. The student's *T*-test was employed to assess the differences in pre- and postoperative creatinine and haemoglobin levels. ANOVA and Pearson's correlation were performed to assess postoperative findings and tumour characteristics. All tests were two-sided with a statistical significance set at *p* < 0.05.

### 2.1. Surgical Technique

**Figure 1:**
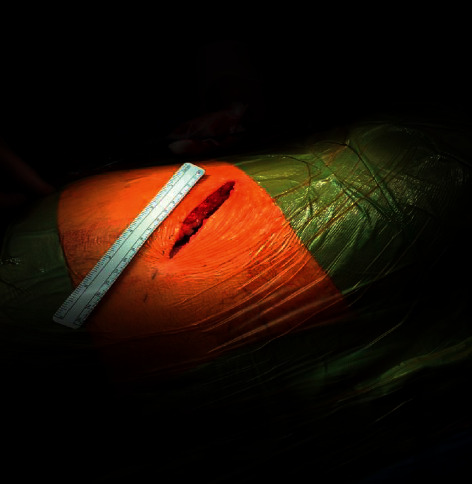
8 cm supra 12th rib miniflank incision.

**Figure 2:**
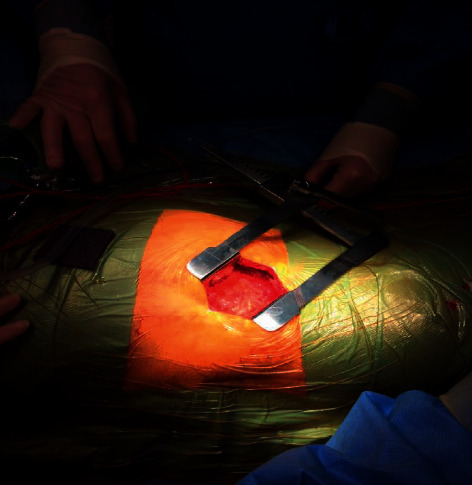
Gerota fascia exposed.

**Figure 3:**
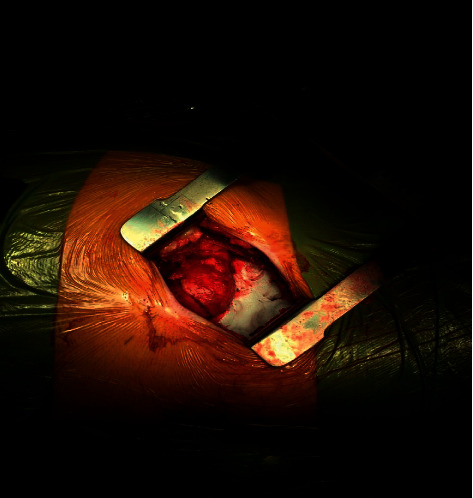
Kidney exposed: tumor delivered high in the wound.

**Figure 4:**
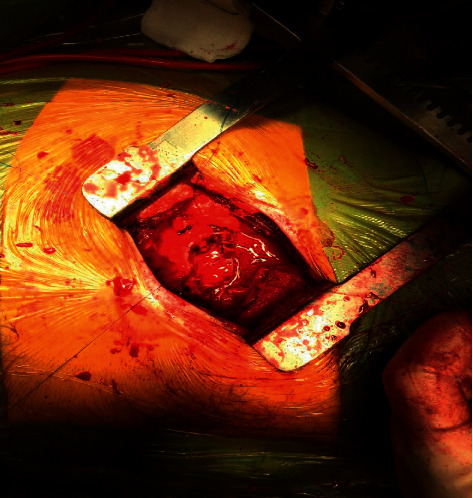
Bleeding vessels tied with polyfilament.

Under general anesthetic, all patients have a ureteric stent inserted prior to undergoing a partial nephrectomy. Patients are placed in a lateral decubitus position, and a supra 12th rib flank incision of 6–8 cm is made (Figure 1) and used to access the retroperitoneum (Figure 2). The kidney is exposed, and a number of small swabs are placed underneath the kidney to deliver the tumour as high as possible in the wound (Figure 3). This is a zero ischaemic technique with no clamping of the renal vessels. The tumour and a suitable margin of paratumour tissue (5–10 mm) is demarcated with the use of diathermy and the lesion resected from the kidney. Any bleeding vessels are tied with polyfilament sutures (Figure 4) and any opening made in the collecting system is closed with monofilament suture material. No bolster or haemostatic patch is applied, and an argon laser is used to aid in coagulation of the tumour bed. The perirenal fat is suitably replaced around the kidney and closed in layers, and no drain is placed as part of the enhanced recovery protocol. Apart from a ureteric stent, a ropivacaine local anaesthetic pump is placed underneath the external intercostal muscle and removed at 48 hours. The patients also follow our departmental enhanced recovery protocol.

## 3. Resultsx

Seventy-one partial nephrectomies were performed by a single surgeon from February 2009 to October 2015. None were converted to a radical nephrectomy (see Table 1). Mean operative time was 72 minutes (range 30–250 minutes), and mean estimated blood loss was 608 ml (range 100–2500 ml) with one patient (1.4%) receiving a blood transfusion intraoperatively. Mean tumour size was 3.69 cm (range 1.8–7.1 cm). The mean pre- and postoperative haemoglobin levels were 144 and 112 g/l (*p*=0.19) with a mean drop in haemoglobin levels of 31 g/l. The mean pre- and postoperative creatinine levels were 82 and 103 Umol/L (*p*=0.07) with a mean increase of 20 Umol/L.

There were 8 (11.3%) Grade 2 (Clavien–Dindo) complications and 1 (1.4%) major complication (Clavien IIIa). Histology confirmed 24 benign lesions and 47 malignant lesions, 46 cT1a lesions, 24 cT1b lesions, and 1 cT2 lesion. Median follow-up was 38 months with no local recurrence or progression of disease with 5 patients (7.0%) having a positive margin. 15 patients (21.1%) had a low renal nephrometry score, 26 patients (36.6%) had an intermediate score, and 30 patients (42.3%) had a high score. There was no significant correlation between renal score and haemoglobin change, estimated blood loss, tumour size, operative time, or creatinine increase postoperatively.

## 4. Discussion

Open PN has become the standard surgical approach for SRMs and cT1b renal lesions. Due to the increase in diagnosis of SRMs in clinical practice, the open PN technique remains an important technique for surgical oncologists [2]. All patients having open partial nephrectomy had a ureteric stent inserted to facilitate urinary drainage which is of particular importance in cases where the collecting system was opened during excision of tumour. Placement of a ureteric stent also makes the insertion of an abdominal drain unnecessary and prevents associated complication, e.g., pain, dislodgement, and or premature removal of drain. The main risk of a zero ischaemic PN is haemorrhage [12]. In our series, the EBL was 608 mls with no cases of urinomas. Clamping the renal vessels could have dramatically reduced the bleeding; however, this may increase the risk of long-term renal impairment. One of our patients required a transfusion (1.4%). The literature suggests that the rate of transfusion for PN varies between 4 and 20% [13]. A recent publication of a series of 40 open zero ischaemia PN had a comparable estimated blood loss of 521 ml [13]. There are laparoscopic and robotic series that employed a zero ischaemic technique with a lower EBL (206 ml); however, they had quite a high transfusion rate of 21% and longer mean operating time of 4.4 hours and a range of up to 8 hours [14].

Similarly, one study in 2018 of 308 patients in a single-centre undergoing robotic off-clamp partial nephrectomies had an overall transfusion rate of 6.2% (*n* = 19). Clavien–Dindo ≥3 grade complications occurred in 4 (1.3%) of patients. Mean EBL was 280 mls (range 50–800) which is less compared to our cohort, and mean creatinine increase was 13% at discharge [15]. Another study looking at laparoscopic and robot-assisted partial nephrectomy with off-clamp technique and controlled hypotension followed 60 patients with a median tumour size of 3.6 cm. Median EBL was 200 ml (range 30–700 ml) with 4 patients (6.6%) requiring postoperative blood transfusions. Due to controlled hypotension, only ASA grade 1-2 patients were selected in this cohort [16].

We always attempted to remove the lesion with a 5–10 mm paratumour safety margin. Given limited anatomical landmarks, this provides a challenge for complex lesions whilst striving to preserve as much benign renal parenchyma as possible. Median follow-up was 38 months with no local recurrence or progression of disease with 5 patients having a positive margin (7%) which is a similar rate to a UK national partial nephrectomy audit of 7% [17]. Other international publications have positive surgical margin rates of 0–31% [18, 19].

Another study from the UK employed an open partial nephrectomy technique. Although they used soft bowel clamps to the kidney to reduce bleeding, with an estimated blood loss of 400 ml, but a statistically significant deterioration in postoperative renal function. In this series of 100 patients, there was 1 case with a positive surgical margin, and there were 2 cases of tumour recurrence [20]. In our series, there is neither a clinically nor a statistically significant change in postoperative renal function or haemoglobin levels.

From a medico-economical perspective, an open PN would have significant cost savings relative to a robotic approach due to the financial outlay necessary to purchase the technology, which may only be cost-effective in large, high volume centres. Our enhanced recovery pathway also aids in our ability to efficiently and safely discharge patients and keep length of stay to a minimum. Patient education is crucial for an efficient discharge and requires regular communication with the patient and family pre-, peri-, and postoperatively to maintain compliance with the enhanced recovery pathway.

The debate will continue as to whether the optimum option for patients requiring a PN is a robotic, laparoscopic, or an open approach. Conventional wisdom has taught us that there are many complex tumours that are technically easier and safer to perform with an open approach. However, surgeon skill and experience as well as an efficient theatre team are paramount for a safe, efficient, open PN regardless of the technique employed.

In the era of robotic and laparoscopic techniques for PN, our supra 12th rib miniflank incision is a suitable alternative for high risk tumours. The 6–8 cm incision leaves the patients with a small, cosmetically acceptable scar. This remains a suitable option for patients who are managed in centres which are not in a position to offer patients a laparoscopic or robotic approach.

## 5. Conclusion

Our results demonstrate that a supra 12th rib miniflank incision open partial nephrectomy with zero ischaemia time for small renal masses have satisfactory outcomes with preservation of renal function and a small satisfactory cosmetic scar. A minimal access open partial nephrectomy is a fast, safe, and technically easy approach for small renal masses and remains an important and viable treatment option.

## Figures and Tables

**Table 1:** Demographics, tumour characteristics, renal nephrometry score, estimated blood loss, and complications.

Benign		Duration	
Oncocytoma	8	<1 hr	4
Cystic nephroma	5	1-2 hrs	51
Renal cyst	9	2-3 hrs	14
Angiomyolipoma	2	3-4 hrs	1
	**24**	<4 hrs	1
Malignant		Mean	72 minutes
Clear cell	39	Estimated blood loss	
Chromophobe	6	<500 ml	42
Papillary type 1	2	5–1000	23
	**47**	1000–2500	6
Fuhrman/nuclear grade		Mean	608 ml
Grade 1	10	Clavien–Dindo complications	
Grade 2	24	Grade 2	8
Grade 3	13	Grade 3a	1
Grade 4	0	Total	**9**
	**47**	Size	
RENAL nephrometry score		<4 cm	46
4–6	15	4–7 cm	24
7–9	26	>7 cm	1
10–12	30	Mean	3.69 cm

## Data Availability

Access to the data set is restricted as it is currently being used for other research/publications. However relevant data sets might be made available on request.
